# Alleviating Pregastroscopy Anxiety Using Mobile Social Media Application

**DOI:** 10.3389/fmed.2022.855892

**Published:** 2022-06-22

**Authors:** Dan Lu, Jing-Hua Wang, Chao Lu, Zheng-Lv Liu, Ajay Jain, Feng Ji, Qing Gu

**Affiliations:** ^1^Department of Endoscopy Center, The First Affiliated Hospital, College of Medicine, Zhejiang University, Hangzhou, China; ^2^Department of Gastroenterology, The First Affiliated Hospital, College of Medicine, Zhejiang University, Hangzhou, China; ^3^Department of Anesthesiology, The First Affiliated Hospital, College of Medicine, Zhejiang University, Hangzhou, China; ^4^Meridian Medical Group, Indiana University Health Methodist Hospital, Indianapolis, IN, United States

**Keywords:** gastroscopy, anxiety, social media, mobile application, patient cooperation, tolerance

## Abstract

**Aim:**

The research aimed to study the effect of using WeChat (a mobile social media application) on pregastroscopy anxiety and the cooperation of patients with different coping styles.

**Methods:**

In order to decrease patients' pregastroscopy anxiety and improve the tolerance of unsedated gastroscopy, WeChat, a widely used mobile social media application, was applied to provide information prior to their endoscopic procedure. Two hundred and thirty patients who underwent initial unsedated gastroscopy in a large teaching hospital in China were classified into two groups based on their coping style: information seekers or information avoiders, using the Information Subscale of the Krantz Health Opinion Survey (KHOS-I). Each of the two groups was prospectively randomly assigned to either receiving the brochure information or conjunctive interactive WeChat-delivered information of gastroscopy. To measure the level of state anxiety, the State Anxiety Scale of Spielberg's State-Trait Anxiety Inventory questionnaire was used. State anxiety, blood pressure and heart rate were measured at enrollment, upon arrival, and before gastroscopy.

**Results:**

Information seekers and avoiders who received information from the brochure and the WeChat platform experienced significantly less state anxiety upon arrival and before gastroscopy. Furthermore, information seekers who received information from the conjunctive WeChat platform had lower frequency of retching, lower scores of nausea and bloating, and better tolerance. Information avoiders who received information from the conjunctive WeChat platform had lower frequency of retching, lower scores of discomfort while swallowing the scope and nausea, and better tolerance. However, we found the percentage of information seekers who preferred no WeChat-delivered pregastroscopy information is greater than WeChat-delivered information at the initial questionnaire. No significant difference was found in blood pressure or heart rate upon arrival and before gastroscopy.

**Conclusions:**

Although people preferred no WeChat-delivered pregastroscopy information, the provision of gastroscopy information through a mobile social media application, such as WeChat, could significantly reduce patients' pregastroscopy anxiety, lower the frequency of retching, reduce the scores of nausea and bloating, and improve tolerance for information seekers. In addition, it could lower the frequency of retching, reduce the scores of discomfort while swallowing the scope and its concurrent nausea, and improve tolerance for information avoiders.

## Introduction

Gastric cancer has become the second leading cause of death worldwide. Furthermore, incidence rates are highest in Eastern and Central Asia and confer a higher mortality rate there than in other nations (1). One well-documented method for gastric cancer prevention is *via* endoscopic screening in the asymptomatic population (2). Despite this globally well-known procedure, patients quite often perceive this procedure as uncomfortable and/or possibly embarrassing and may have concerns with potential exam results. These feelings about a commonplace procedure are generated from limited information, distress caused by perceived fear of discomfort, and an unfamiliarity with the process (3). Such preoccupations produce burdensome anxiety (4). As the need for these procedures increases, the process is dictated by direct referral and so the chance to meet the endoscopist in advance of the procedure is often bypassed, and also the ability of physicians to detect and gauge patient anxiety has proven less than adequate (5, 6).

Furthermore, procedural anxiety could affect patients' satisfaction and impede patient compliance with this routine procedure, and make it more difficult for them to tolerate gastroscopy (7, 8). There are instances whereby the stomach cannot be thoroughly examined which can be attributed to poor patient cooperation. In addition, the lack of patient cooperation amplifies the possibility of endoscopic complications and the miss rate of significant gastric lesions (9).

Recently, alternative methods, including tools like booklets, cartoons, and short message services (SMS) (10–12), have been used to relieve patients' stress and improve patient cooperation (13). Even more recently, smart phone-based strategies have been developed to help patients get prepared (14–21).

One critical method to consider when dealing with patients is their coping styles. In treatment, there may be information seekers, those who like to gather as much information as possible about the illness and/or procedure to make it more predictable and controllable, or they may be information avoiders, those who like to avoid the stressful situation and distract themselves from any threat-relevant information, siding toward unpredictability. Taking patients' coping styles into consideration could reduce procedural anxiety more effectively (22, 23). This is even as important as the content of the illness itself.

Liu, et al. reported that the provision of sensory information could reduce patients' pregastroscopy anxiety significantly, regardless of patients' information coping style (24). Morgan, et al. found information congruent with coping style reduced anxiety and observed behavioral indices of colonoscopy pain (25). In a study by Yang, et al., it was demonstrated that pregastroscopy anxiety was an independent predictor of severe discomfort and poor tolerance in patients undergoing unsedated gastroscopy (26). Kang, et al. claimed instruction *via* a mobile social media application, in conjunction with standard instruction, improves the adequacy of bowel preparation for colonoscopy (16). However, to date, few studies have been conducted to identify the effect of interactive information delivery *via* mobile social media application on the pregastroscopy anxiety of different coping styles, patient cooperation and tolerance during gastroscopy.

WeChat is the most widely used multipurpose social media platform in China, which is integrated with messaging, voice and video calls, and other services. The number of daily active WeChat users is estimated to be around 1 billion and the popularity of WeChat stems from its instant messaging and interaction function (27). WeChat could also provide a platform for medical professionals to more effectively clarify and reassure gastroscopy information. Through WeChat, patients can raise their concerns in complete privacy.

In the study we performed a prospective, randomized, controlled trial to compare the effect of pregastroscopy anxiety, and patient cooperation of different coping styles receiving gastroscopy informational brochure with interactive WeChat-delivered information vs. the informational brochure only. We tested the hypothesis that information delivered by the WeChat reduces pregastroscopy anxiety, improves patient cooperation, and tolerance for information seekers. And we also tested the hypothesis that the percentage of information seekers who preferred WeChat-delivered pregastroscopy information is greater than no WeChat-delivered information.

## Materials and Methods

### Study Design

This prospective, single-blinded, randomized, controlled study was conducted in the Endoscopy Center in a large teaching hospital in southeast China from 28 June to 8 August 2020. The institutional review board approved the study protocol and informed consent form (Number of Approval: IIT20200203A-R1). This study has been registered at www.Chictr.org.cn (ChiCTR2000034213).

All the patients received pregastroscopy information in the form of an official brochure when scheduling the gastroscopy. The brochure was handed out by two designated nurses who were not involved in gastroscopy and blinded to the randomization. The informational brochure was written in simple words and illustrated by animated pictures.

The patients were classified into two groups based on coping style: information seekers or information avoiders using the Information Subscale of the Krantz Health Opinion Survey (KHOS-I) (24, 28). Each group was randomly assigned by computer-generated random numbers to receive informational brochure only (control group) or both informational brochure and interactive information delivered by the mobile application, WeChat (WeChat group).

Both information seekers and information avoiders allocated to the WeChat portion of the study were invited to get access to the WeChat (Hospital official account: Endoscopy Center) on the day of the gastroscopy scheduling by two trained nurses who were not involved in data collection. In addition to receiving the brochure, they also received the same information delivered by WeChat (Hospital official account: Endoscopy Center). Possible interaction was the only difference between the control and WeChat group of both the information seekers and avoiders. Otherwise, all the information across all four groups was the same.

One nurse practitioner logged in to the WeChat platform using the official account between 4 and 6 p.m. daily to explain the brochure information which some patients could not fully understand by themselves. The nurse practitioner was trained to use therapeutic communication skills to address patients' concerns and give consistent answers to the same question.

All patients and their families were instructed not to disclose that they had access to the WeChat-delivered information, to endoscopists, medical staff, or other patients. State anxiety was assessed by the State Anxiety Scale of Spielberg's State Anxiety Inventory (29). Blood pressure, and pulse were measured at enrollment, upon arrival, and before gastroscopy by a designated nurse that was not involved in the procedure of gastroscopy and blinded to the randomization.

The gastroscopy was performed by 2 professional endoscopists with a minimum experience of 5,000 gastroscopies. The gastroscope (GIF-HQ290; Olympus), Radial Jaw (Boston Scientific), and mouthpiece (MB-142; Olympus) were used for each procedure and a topical anesthetic was applied to anesthetize the throat to suppress the gag reflex. The setup of the endoscopy room is unified.

Patient cooperation, patient discomfort, and tolerance were recorded by trained nurses. Belching, retching, and coughing were the main manifestations of poor cooperation (9). And the visual analog scale (VAS) was used to measure patients' discomfort during the procedure (30–33), including the scope passing through the throat, nausea, abdominal pain, and bloating. Patients were asked to rate the severity of their symptoms from “0–10,” with “0” being “I felt absolutely comfortable during the procedure” and “10” being “I was suffering to death during the procedure.” And patient tolerance was recorded by the answer to the question of the acceptability of unsedated gastroscopy after the procedure (Easy, A little difficult, Very difficult, and Cannot endure). The endoscopists and trained nurses were blinded to the participants.

### Study Participants

Participants were from 18–70 years of age and underwent their initial gastroscopies in regular health screening, without any former experience of colonoscopy or bronchoscopy. Patients were considered to meet the inclusion criteria in the study if: They were mentally alert and able to communicate freely, underwent initial, unsedated gastroscopy as outpatients, and had access to WeChat themselves or through close family members. Patients were excluded if they suffered from severe cardiopulmonary disease, underwent emergency gastroscopy, had impaired consciousness or impaired hearing, were mentally distressed or underwent other invasive procedures on the same days, such as colonoscopy contrast enhanced CT, and an ultrasound-guided fine needle aspiration, etc. No sample sizes were performed a priori, as it is difficult to find other studies presenting data which could be used to estimate variance and effect size.

### Assessment Methods

Participants' personal characteristics were collected by a questionnaire including their gender, age, education level, employment status, income level, and family gastric and/or esophageal cancer history, their preferences of receiving information *via* WeChat or the brochure, their knowledge about gastroscopy, and days of waiting for gastroscopy upon enrollment.

The KHOS-I subscale was used to determine patients' coping style by answering “Yes/No” questions relating to their preference for information, their desire to be involved in medical decision-making and their own wish to raise questions about the procedure (34).

Furthermore, subjects completed the Spielberger State Anxiety Inventory, which measures degrees of anxiety (e.g., feelings of tension, apprehension, and nervousness.). It consists of 20 statements and each statement has “1–4” points, with “4” indicating greatest anxiety (35).

All the information distributed *via* the brochure and through the WeChat (Hospital official account: Endoscopy Center), was collected based on information guides (Endoscopy (Upper GI) from (36)) aga gi patient center and Understanding Upper Endoscopy from ASGE, and from the suggestions of endoscopists, nurses and patients. It consists of five parts, which are detailed in Supplementary Material 1.

### Statistical Analysis

The independent samples *t*-test for age, days of waiting for gastroscopy, and Chi-square test for gender, education level, employment status, income level, and family history of gastric and/or esophageal cancer, preferences of receiving information *via* WeChat or not, preferences of receiving information *via* the brochure or not, knowledge about gastroscopy, were used to determine whether there were baseline differences. *P* < 0.05 was used as the significance level in this study.

An independent sample *t*-test for state anxiety score, blood pressure, and pulse, frequency of coughing, belching, retching, and scores of discomfort while swallowing the scope, nausea, abdominal pain, and bloating, and Chi-square test for tolerance, was used to find any significant differences between the control group and the WeChat group in information seekers and information avoiders. Multiple regression analysis was used to determine the factors predicting state anxiety. The data were analyzed by using Statistical Package for Social Sciences (SPSS) version 25.

## Results

### Patient Baseline Characteristics

**Figure 1:**
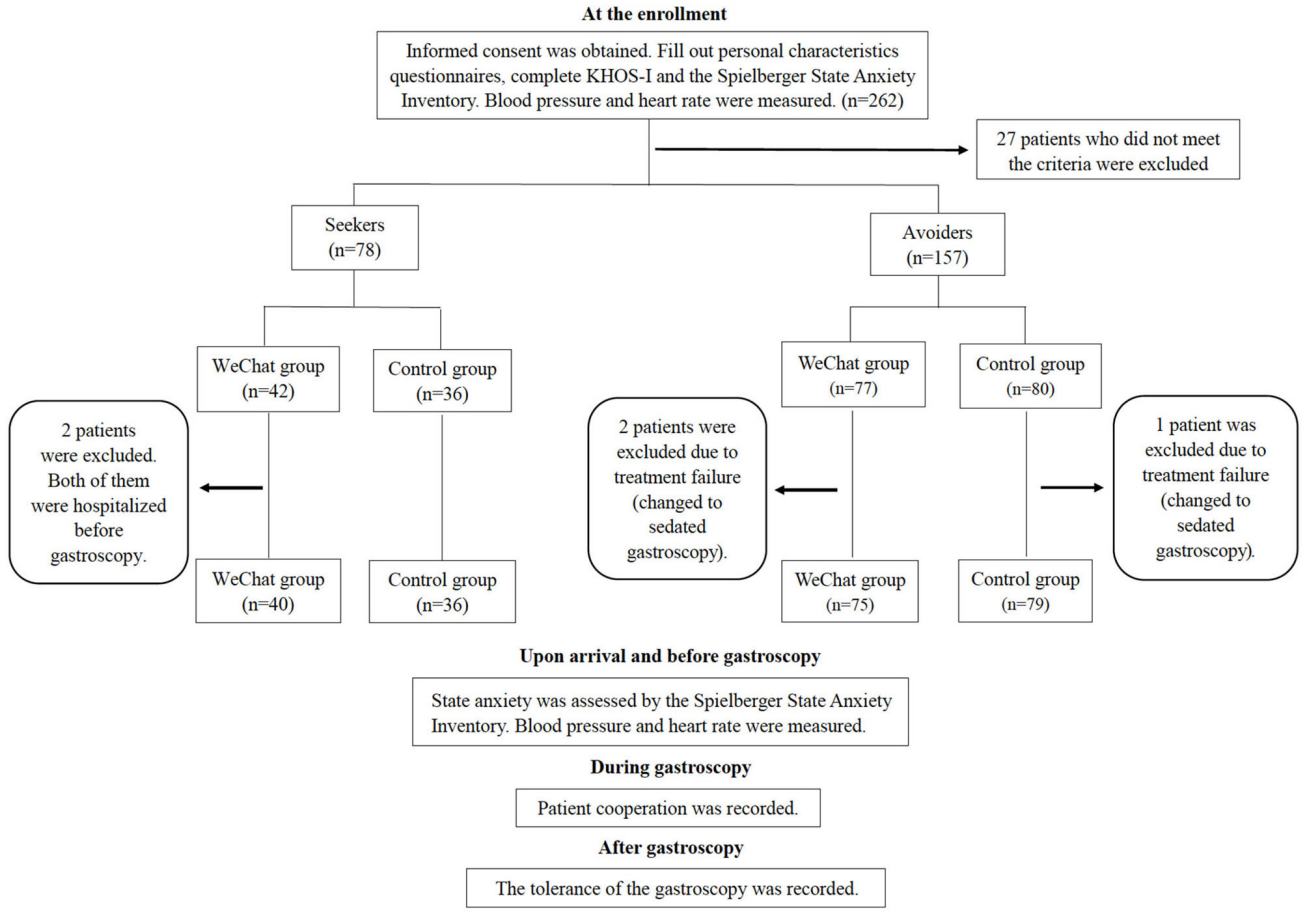
The flowchart of procedure. KHOS-I indicates Krantz Health Opinion Survey.

Two hundred and sixty-two patients were screened in total. Twenty-seven patients (one patient with severe cardiopulmonary disease, 10 patients who underwent emergency gastroscopy, four patients with psychiatric mental health problems, 10 patients who made the appointment of colonoscopy and two patients who made the appointment of contrast enhanced CT on the same day with gastroscopy), who did not meet the criteria were excluded. Two patients of information seekers from the WeChat group who were hospitalized before gastroscopy were excluded. This study also excluded three cases of treatment failure: Two patients of information avoiders from the WeChat group and one patient of information avoiders from the control group who could not tolerate the procedure and changed to the sedated procedure. A total of 230 participants (76 Seekers and 154 Avoiders) completed all the questionnaires (Response Rate = 97.9%) (Figure 1).

As shown in Table 1, there were no significant differences among the measured patient baseline parameters. However, Table 1 shows that greater percentage of participants in all groups preferred pregastroscopy information “brochure” over “no brochure information,” and greater percentage of participants preferred “no WeChat pregastroscopy information” over “WeChat pregastroscopy information.”

**Table 1:** Baseline characteristics of patients undergoing gastroscopy included in the study.

	**Seekers**		***P***	**Avoiders**		***P***
	**WeChat group**	**Control group**		**WeChat group**	**Control group**	
	**(*n* = 40) *N* (%)**	**(*n* = 36) *N* (%)**		**(*n* = 75) *N* (%)**	**(*n* = 79) *N* (%)**	
Sex						
Male	23 (57.50)	17 (47.22)	0.370	48 (64.00)	43 (54.43)	0.227
Female	17 (42.50)	19 (52.78)		27 (36.00)	36 (45.57)	
Education level						
Primary school	5 (12.50)	3 (8.32)	0.240	7 (9.33)	7 (8.86)	0.113
high school	9 (22.50)	11 (30.56)		30 (40.00)	24 (30.38)	
Undergraduate/Da zhuan	18 (45.00)	20 (55.56)		35 (46.67)	36 (45.57)	
Master's degree or above	8 (20.00)	2 (5.56)		3 (4.00)	12 (15.19)	
Employment status						
Employed	27 (67.50)	27 (75.00)	0.472	46 (61.33)	56 (70.89)	0.210
Unemployed	13 (32.50)	9 (25.00)		29 (38.67)	23 (29.11)	
Family income						
≤ 4,000 ($565)	11 (27.50)	8 (22.22)	0.643	18 (24.00)	19 (24.05)	0.953
4,000–8,000 ($565–$1,130)	8 (20.00)	10 (27.78)		22 (29.34)	22 (27.85)	
8,000–10,000 ($1,130–$1,413)	9 (22.50)	5 (13.89)		16 (21.33)	15 (18.99)	
10,000 ($1,413) and above	12 (30.00)	13 (36.11)		19 (25.33)	23 (29.11)	
Family gastric and/or esophageal cancer history						
Yes	4 (10.00)	7 (19.44)	0.243	14 (18.67)	9 (11.39)	0.206
No	36 (90.00)	29 (80.56)		61 (81.33)	70 (88.61)	
Preference of receiving information *via* WeChat						
Yes	7 (17.50)	4 (11.11)	0.429	12 (16.00)	12 (15.19)	0.890
No	33 (82.50)	32 (88.89)		63 (84.00)	67 (84.81)	
Preference of receiving information *via* brochure						
Yes	25 (62.50)	29 (80.56)	0.083	49 (65.33)	44 (55.70)	0.222
No	15 (37.50)	7 (19.44)		26 (34.67)	35 (44.30)	
Knowledge about gastroscopy						
None	17 (42.50)	19 (52.78)	0.370	39 (52.00)	37 (46.84)	0.809
A little	23 (57.50)	17 (47.22)		35 (46.67)	41 (51.90)	
A lot	0 (0)	0 (0)		1 (1.33)	1 (1.26)	
Age	35.10 ± 13.55	37.30 ± 12.05	0.463	38.09 ± 13.01	35.10 ± 11.52	0.134
Days of waiting for gastroscopy	3.48 ± 1.95	3.31 ± 2.21	0.724	3.33 ± 2.40	3.51 ± 2.98	0.693

“*Age” and “Days of waiting for gastroscopy” are presented as mean ± SD*.

*WeChat group: Received Conjunctive WeChat-delivered information*.

*Control group: Received the brochure information*.

As shown in Table 2, no significant difference was found in anxiety level, blood pressure (BP), heart rate (HR), upon enrollment at baseline for information seekers and information avoiders, between patients who received gastroscopy information *via* the brochure and those who received information *via* brochure as well as the WeChat platform. There was no significant difference in endoscopist distribution between groups (Supplementary Tables S1, S2).

**Table 2:** Anxiety level at baseline, upon arrival and before gastroscopy, and patient cooperation and tolerance by information seekers and avoiders in each of the two information groups.

	**Seekers**		***P***	**Avoiders**		***P***
	**WeChat group**	**Control group**		**WeChat group**	**Control group**	
**State anxiety**						
Baseline	39.05 ± 6.57	36.81 ± 8.24	0.191	37.87 ± 10.54	36.44 ± 8.56	0.358
Upon arrival	34.80 ± 7.04	40.97 ± 9.15	0.001	32.17 ± 7.90	42.73 ± 7.83	P <0.001
Before gastroscopy	31.98 ± 7.26	40.78 ± 8.51	P <0.001	30.53 ± 12.97	43.24 ± 7.71	P <0.001
**SBP**
Baseline	129.50 ± 14.29	127.64 ± 14.09	0.570	128.28 ± 15.57	127.28 ± 12.53	0.660
Upon arrival	130.85 ± 15.59	128.89 ± 13.99	0.567	129.08 ± 14.73	128.72 ± 15.57	0.884
Before gastroscopy	126.35 ± 14.71	125.86 ± 15.85	0.889	123.27 ± 15.19	126.44 ± 15.06	0.195
**DBP**						
Baseline	80.85 ± 9.84	79.58 ± 11.18	0.601	78.88 ± 9.35	78.16 ± 9.89	0.646
Upon arrival	80.43 ± 8.58	81.83 ± 11.95	0.554	79.72 ± 9.83	78.99 ± 11.66	0.675
Before gastroscopy	77.05 ± 9.37	78.19 ± 11.75	0.638	77.65 ± 10.54	78.03 ± 11.36	0.834
**HR**						
Baseline	86.73 ± 12.17	82.72 ± 9.46	0.117	83.77 ± 13.50	82.43 ± 13.07	0.531
Upon arrival	88.75 ± 15.29	90.14 ± 17.04	0.709	87.28 ± 13.31	89.59 ± 14.80	0.310
Before gastroscopy	83.13 ± 13.08	88.14 ± 15.43	0.130	83.72 ± 14.39	86.61 ± 15.56	0.234
Coughing	0.43 ± 0.87	0.75 ± 1.23	0.184	0.65 ± 1.37	0.89 ± 1.22	0.267
Belching	1.15 ± 1.23	1.58 ± 1.66	0.198	1.23 ± 1.35	1.68 ± 1.71	0.068
Retching	1.33 ± 1.44	4.58 ± 5.23	P <0.001	1.43 ± 1.75	2.85 ± 2.50	P <0.001
Discomfort swallowing the scope	3.10 ± 1.52	4.11 ± 2.81	0.051	2.83 ± 2.24	3.78 ± 2.45	0.012
Nausea	3.30 ± 1.77	5.19 ± 2.25	P <0.001	3.40 ± 2.39	4.91 ± 2.40	P <0.001
Abdominal pain	0.13 ± 0.40	0.36 ± 0.68	0.067	0.11 ± 0.39	0.23 ± 0.58	0.130
Bloating	0.13 ± 0.40	0.44 ± 0.81	0.030	0.16 ± 0.52	0.3 ± 0.77	0.180
**Tolerance**						
Easy	5 (12.50)	1 (2.78)	0.050	16 (21.33)	3 (3.80)	P <0.001
A little difficult	30 (75)	18 (50)		46 (61.34)	37 (46.83)	
Very difficult	5 (12.50)	14 (38.89)		12 (16)	36 (45.57)	
Cannot endure	0 (0)	3 (8.33)		1 (1.33)	3 (3.80)	

*Measured values are presented as mean ± SD or number (percentage)*.

*DBP, diastolic blood pressure; SBP, systolic blood pressure; HR, heart rate*.

*WeChat group: Received Conjunctive WeChat-delivered information*.

*Control group: Received the brochure information*.

*Systolic blood pressure measured in mm Hg*.

*Diastolic blood pressure measured in mm Hg*.

*Heart rate measured in bpm*.

After receiving the intervention, the state anxiety score upon arrival (*P* = 0.001) and before gastroscopy (*P* < 0.001) from the WeChat group of information seekers, and the state anxiety score upon arrival (*P* < 0.001) and before gastroscopy (*P* < 0.001) from the WeChat group of information avoiders, all significantly declined.

In our study, we have two information avoiders from the WeChat group and one information avoider from the control group who could not tolerate the procedure and changed to the sedated procedure. The anxiety levels of the two information avoiders from the WeChat group (35 at baseline, 30 upon arrival, and 39 before gastroscopy; 31 at baseline, 28 upon arrival, and 35 before gastroscopy) showed no significant difference from their group anxiety levels. The anxiety level of the information avoider from the control group (34 at baseline, 40 upon arrival, and 42 before gastroscopy) shows no significant difference from their group anxiety levels either.

No significant difference was found in BP and HR upon arrival and before gastroscopy for information seekers or information avoiders, between those who received gastroscopy information *via* the brochure and those who received information *via* brochure, as well as the WeChat platform.

Compared to information seekers who received information from the brochure only, those who received information from the WeChat and the brochure, had lower frequency of retching (*P* < 0.001), lower scores of nausea (*P* < 0.001), and bloating (*P* < 0.05), and better tolerance (*P* < 0.001).

In contrast to information avoiders who received information from brochure only, those who received information from WeChat and the brochure, had lower frequency of retching (*P* < 0.001), lower scores of Discomfort while swallowing the scope (*P* < 0.05), and nausea (*P* < 0.001), and better tolerance (*P* < 0.001).

Women have been found to have higher anxiety score than men at the baseline when scheduling the procedure (*P* < 0.05), but no significant differences in anxiety score were found upon arrival and before the gastroscopy between women and men (*P* > 0.05) (Table 3).

**Table 3:** Predictors of anxiety level of patients undergoing gastroscopy at three different stages.

**Predictor variables**	**Anxiety baseline**			**Anxiety upon arrival**			**Anxiety before gastroscopy**		
	***B***	**Beta**	***P***	***B***	**Beta**	***P***	***B***	**Beta**	***P***
Gender	2.873	0.160	0.020	2.226	0.120	0.077	2.697	0.118	0.082
Age	0.036	0.050	0.570	0.023	0.032	0.717	−0.009	−0.010	0.908
Education level	0.200	0.018	0.845	0.784	0.069	0.453	−0.48	−0.034	0.709
Employment status	−1.125	−0.059	0.398	−2.907	−0.148	0.034	−1.561	−0.065	0.352
Income level	−0.385	−0.05	0.506	−0.581	−0.073	0.326	−0.039	−0.004	0.958
Family cancer history	0.984	0.039	0.572	2.259	0.088	0.205	4.239	0.133	0.054
Days of waiting for gastroscopy	−0.050	−0.014	0.835	0.118	0.032	0.633	0.071	0.016	0.816
Preference of receiving information *via* WeChat	−2.038	−0.082	0.324	−0.712	−0.028	0.736	0.451	0.014	0.862
Preference of receiving information *via* brochure	−0.020	−0.001	0.990	0.255	0.013	0.868	2.777	0.118	0.142
Knowledge about gastroscopy	−0.003	0	0.998	0.323	0.018	0.786	2.360	0.108	0.109
*R^2^*	0.044			0.053			0.059		
*R*	0.209			0.229			0.242		
*F*	0.998		0.446	1.217		0.281	1.362		0.200

## Discussion

This study found that the anxiety state score improved for information seekers and information avoiders who received information from the brochure as well as the WeChat platform compared to the informational brochure only. This finding was found both upon arrival and before the gastroscopy procedure.

Information seekers who received information from the brochure and the WeChat platform had less frequency of retching, lower scores of nausea, bloating, and better tolerance than information seekers receiving information only from the brochure.

Information avoiders who received information from the brochure and the WeChat platform had less frequency of retching, lower scores of discomfort while swallowing the scope, and nausea, and better tolerance, compared to information avoiders receiving information from the brochure only.

These results support the conclusion that the delivery of pregastroscopy information though mobile social media app could significantly reduce patients' pregastroscopy anxiety no matter the patients' information coping style. Furthermore, it could improve patient cooperation and tolerance.

According to previous studies, Vukmir, et al. reported that a computer printout, like a brochure, does not help most patient comply with the physician's instructions (37). Abbott reported that poor understanding of the procedure may result in lack of patient cooperation (31).

Online interactive guidance conveys a more personalized set of instructions, making them more relevant to the patient (38). Professional communication provided the patients with reassurance and clarity, and helped remove the uncertainty for those who were concerned about their lack of understanding of the procedure.

These conclusions were consistent with some previous studies. Sewitch, et al. reported that a user-centered smart phone application has the benefit of broadening the patient community, educating patients with comprehensive information, and improving patient cooperation (39). Kang, et al. demonstrates that information delivered by the smart phone application WeChat could improve bowel preparation of colonoscopy and patient compliance (16). Vliet, et al. concluded that medical personnel provides invaluable guidance through coaching when preparing patients for gastrointestinal endoscopy (40). Online coaches through a smart phone application, such as WeChat, help fill the gap when patients leave the hospital. Smart phones help patients cope better (14–21). Liu, et al. reported that the state anxiety score significantly declined after the intervention of sensory information for information seekers and information avoiders (24).

However, some studies showed inconsistent outcomes. Morgan, et al. discovered in their anxiety and pain study for patients undergoing initial colonoscopy that patients who received information congruent with coping style experienced less state anxiety, whereas those who received information not congruent with their coping style maintained the same anxiety level (25). There are essential differences between colonoscopy and gastroscopy, even though they are both gastrointestinal endoscopy procedures. Colonoscopy is generally regarded to be a painful procedure (41), and a considerable proportion of patients experience pain (42). However, only a few patients complain of pain and bloating in gastroscopy. The potential for bloating and especially for pain may frighten information avoiders.

In this study, we found that the percentage of people who preferred brochure pregastroscopy information is greater than no brochure information in all groups, and the percentage of people who preferred no WeChat-delivered pregastroscopy information is greater than WeChat-delivered information in all groups, which is inconsistent with our hypothesis that the percentage of information seekers who preferred WeChat-delivered pregastroscopy information is greater than no WeChat-delivered information. The cause could be attributed to unfamiliarity with WeChat as a mode of education. There is no up to date interactive information regarding pregastrscopy patient education delivered *via* mobile application.

Contrary to our assumptions, information-avoiders also had reduced anxiety level for receiving information through WeChat, which is in contrast to previous studies and original theory (22, 23, 25, 43), in which avoiders were associated with lower demand for information. However, Sewitch, et al. reported that the ability to tailor instructions made the smartphone application preferable to other delivery modes (39). An explanation might be that avoiders did not reject formation input through a non-face to face manner. As such, a social media application that comes from a trusted source, is capable of sending timely and tailored messages, provides reassurance, has clear instructions, and is easy to use (39), may benefit information-avoider patients in the future. Furthermore, we provided information *via* WeChat with the intent of soothing and calming, believing it constitutes a less threatening means of communication. Without having to look someone in the eye, avoiders may feel more reassured and relaxed using it.

In accordance with previous investigations, women have been found to have higher anxiety score than men when scheduling the procedure. In the investigation of Ersöz, et al., women scored higher STAI state anxiety scores than men in gastroscopy and colonoscopy (44). Luck, et al. claimed higher anxiety levels in female patients before colonoscopy (45). Liu, et al. reported that gender was a predictor of state anxiety prior to gastroscopy (24). Shafer, et al. reported variables associated with higher anxiety about bowel preparation were female gender (46). Muzzarelli, et al. revealed that women had higher percentile of the state anxiety raw score measured prior to a scheduled endoscopy (47).

Therefore, the difference in which men and women handle information is an essential consideration for healthcare providers and should focus on future studies into the use and effectiveness of social media applications in reducing anxiety in medical procedures such as gastroscopy.

The study's major strength is the prospective randomized single-blind design and use of validated scales to assess the effect of interactive instructions *via* WeChat on patient anxiety toward unsedated gastroscopy. However, our current study has a few limitations. Firstly, the study was performed in a single center and we could focus on a multicenter study in the future to test the outcome. Furthermore, the sedated patients were excluded, which may introduce bias in the state anxiety outcome. Moreover, VAS was applied to measure the patient's discomfort, but one single scale may not be enough. As a result, multi-validated scales should be used to measure patient discomfort in the future. A final limitation is that the study's results may not be widely applicable in countries where unseated gastroscopy is not the norm.

In conclusion, although people prefer to receive information *via* brochure, the provision of the brochure with WeChat-based disseminated information reduced patients' pregastroscopy anxiety no matter their information coping styles. An acceptable and wide-reaching smartphone application may decrease pregastroscopy anxiety, improve patient cooperation, and tolerance.

## Data Availability Statement

The original contributions presented in the study are included in the article/Supplementary Material, further inquiries can be directed to the corresponding authors.

## Ethics Statement

The studies involving human participants were reviewed and approved by the Clinical Research Ethics Committee of the First Affiliated Hospital, College of Medicine, Zhejiang University (Approval Number: IIT20200203A-R1). Written informed consent was obtained from the individual(s) for the publication of any potentially identifiable data included in this article.

## Author Contributions

DL, QG, and FJ conceived of the study. DL, J-HW, and CL collected and analyzed data. DL, Z-LL, and AJ drafted and critically revised the manuscript. All authors contributed to the article and approved the submitted version.

## Conflict of Interest

The authors declare that the research was conducted in the absence of any commercial or financial relationships that could be construed as a potential conflict of interest.

## Publisher's Note

All claims expressed in this article are solely those of the authors and do not necessarily represent those of their affiliated organizations, or those of the publisher, the editors and the reviewers. Any product that may be evaluated in this article, or claim that may be made by its manufacturer, is not guaranteed or endorsed by the publisher.
