# Supplementary material for: Alleviating Pregastroscopy Anxiety Using Mobile Social Media Application
Source: Front Med (Lausanne). 2022 Jun 22;9:855892. doi: 10.3389/fmed.2022.855892 (PMC9258686; doi:10.3389/fmed.2022.855892)
Supplement: Supplementary file 1 [file Table_1.pdf]

Table S1 Baseline characteristics of patients undergoing gastroscopy included in the study

	Endoscopist 1 (n=105) N(%)	Endoscopist 2 (n=125) N(%)	*P*
Sex			
Male	61(58.1)	70(56.0)	0.790
Female	44(41.9)	55(44.0)	
Education Level			
Primary school	11(10.5)	11(8.8)	0.758
high school	35(33.3)	39(31.2)	
Undergraduate/Da zhuan	46(43.8)	63(50.4)	
Master's degree or above	13(12.4)	12(9.6)	
Employment status			
Employed	70(66.7)	86(68.8)	0.778
Unemployed	35(33.3)	39(31.2)	
Family income			
≤ 4000 ($565)	28(26.7)	28(22.4)	0.458
4000-8000($565-$1130)	26(24.8)	36(28.8)	
8000-10000($1130-$1413)	17(16.2)	28(22.4)	
10000($1413) and above	34(32.4)	33(26.4)	
Family gastric and/or esophageal cancer history			
Yes	12(11.4)	22(17.6)	0.199
No	93(88.6)	103(82.4)	
Preference of receiving information via WeChat			
Yes	13(12.4)	22(17.6)	0.357
No	92(87.6)	103(82.4)	
Preference of receiving information via brochure			
Yes	64(61.0)	83(66.4)	0.411
No	41(39.0)	42(33.6)	
Knowledge about gastroscopy			
None	55(52.4)	57(45.6)	0.194
A little	50(47.6)	66(52.8)	
A lot	0(0.0)	2(1,6)	
Age	35.22±12.17	37.45±12.66	

"Age" is presented as mean ± SD.

WeChat group: Received Conjunctive WeChat-delivered information.

Control group: Received the brochure information.

Table S2 Anxiety level at baseline, upon arrival and before gastroscopy, and patient cooperation and tolerance

	Endoscopist 1 (n=105)	Endoscopist 2 (n=125)	*P*
State Anxiety			
Baseline	37.92±9.07	36.99±8.80	0.431
Upon arrival	36.77±9.65	36.74±12.61	0.981
Before gastroscopy	38.22±9.24	37.14±9.12	0.377
SBP			
Baseline	126.83±13.95	129.91±13.93	0.280
Upon arrival	126.78±15.49	130.30±14.29	0.123
Before gastroscopy	123.70±14.78	126.64±15.36	0.143
DBP			
Baseline	77.58±10.68	80.35±9.04	0.340
Upon arrival	77.93±10.42	81.59±10.54	0.090
Before gastroscopy	76.61±11.21	78.73±10.33	0.138
HR			
Baseline	82.64±12.13	84.52±12.93	0.259
Upon arrival	87.76±13.52	89.63±15.70	0.339
Before gastroscopy	84.75±14.22	85.76±15.27	0.605
Coughing	0.80±1.44	0.64±0.99	0.086
Belching	1.47±1.63	1.39±1.42	0.712
Retching	2.31±2.18	2.46±3.55	0.722
Discomfort			
Swallowing the scope	3.32±2.32	3.47±2.38	0.635
Nausea	4.06±2.46	4.29±2.36	0.470
Abdominal pain	0.24±0.63	0.18±0.39	0.230
Bloating	0.23±0.58	0.26±0.72	0.685
Tolerance			
Easy	12(11.4)	13(10.4)	0.992
A little difficult	69(57.1)	71(56.8)	
Very difficult	30(28.6)	37(29.6)	
Cannot endure	3(2.9)	4(3.2)	

Measured values are presented as mean ± SD or number (percentage).

DBP = diastolic blood pressure; SBP = systolic blood pressure; HR=heart rate

WeChat group: Received Conjunctive WeChat-delivered information.

Control group: Received the brochure information.

Systolic blood pressure measured in mm Hg.

Diastolic blood pressure measured in mm Hg.

Heart rate measured in bpm.
