# Supplementary material for: Alleviating Pregastroscopy Anxiety Using Mobile Social Media Application
Source: Front Med (Lausanne). 2022 Jun 22;9:855892. doi: 10.3389/fmed.2022.855892 (PMC9258686; doi:10.3389/fmed.2022.855892)
Supplement: Supplementary file 2 [file Data_Sheet_1.pdf]

SUPPLEMENTARY MATERIAL 1

All the information distributed via the brochure and through WeChat is from the Hospital official account (Endoscopy Center), and was collected on the basis of information guides (Endoscopy (Upper GI) from aga gi patient center and Understanding Upper Endoscopy from ASGE), and from the suggestions of endoscopists, nurses and patients. It consists of five parts:

I. Procedural information explains why the procedure was carried out and a brief introduction to gastroscopy.

II. Preparation for gastroscopy

A. Drug information (stop taking aspirin or any blood thinners at least seven days before gastroscopy. Anti-hypertensive drugs must be taken as usual on the day of gastroscopy, with a maximum of 20-milliliter water.

B. Diet instruction: No more food after dinner on the night before the procedure and no more water after midnight, as gastroscopy is carried out the next morning.

C. Necessary lab results needed to bring along on the day of the procedure.

D. Other attention, like removal of the denture before the procedure

III. Sensory information describes the sensations that patients may expect during the procedure, including what the patients may feel, see, hear, or smell.

IV. Instructions after gastroscopy, including when and where to get the results

V. Information on respiratory activity (breathe in air through the nose and breathe out air through the mouth) was explained in order to alleviate coughing, belching, retching, and vomiting during gastroscopy.
